# Mitogenome Sequencing in the Genus *Camelus* Reveals Evidence for Purifying Selection and Long-term Divergence between Wild and Domestic Bactrian Camels

**DOI:** 10.1038/s41598-017-08995-8

**Published:** 2017-08-30

**Authors:** Elmira Mohandesan, Robert R. Fitak, Jukka Corander, Adiya Yadamsuren, Battsetseg Chuluunbat, Omer Abdelhadi, Abdul Raziq, Peter Nagy, Gabrielle Stalder, Chris Walzer, Bernard Faye, Pamela A. Burger

**Affiliations:** 1 Research Institute of Wildlife Ecology, Vetmeduni Vienna, Savoyenstraße 1, 1160 Vienna, Austria; 2 Institute of Population Genetics, Vetmeduni Vienna, Veterinärplatz 1, 1210 Vienna, Austria; 3 Institute for Molecular Evolution and Development, University of Vienna, Althanstrasse 14, 1090 Vienna, Austria; 4 Department of Biology, Duke University, Durham, NC 27708 USA; 5 Department of Biostatistics, University of Oslo, N-0317 Oslo, Norway; 6 Department of Mathematics and Statistics, University of Helsinki, FIN-00014 Helsinki, Finland; 7 Mammalian Ecology Laboratory, Institute of Biology, Mongolian Academy of Sciences, Peace avenue-54b, Bayanzurh district, Ulaanbaatar, 210351 Mongolia; 8 Laboratory of Genetics, Institute of Biology, Mongolian Academy of Sciences, Peace avenue-54b, Bayanzurh district, Ulaanbaatar, 210351 Mongolia; 9 University of Khartoum, Department for Meat Sciences, Khartoum, Sudan; 10 Lasbela University of Agriculture, Water and Marine Sciences, Regional Cooperation for Development (RCD) Highway, Uthal, Pakistan; 11 Farm and Veterinary Department, Emirates Industry for Camel Milk and Products, PO Box 294239, Dubai, Umm Nahad United Arab Emirates; 12 International Takhi Group - Mongolia, Baigal Ordon, Ulaanbaatar, Mongolia; 13 CIRAD-ES, UMR 112, Campus International de Baillarguet, TA C/112A, 34398 Montpellier, France

## Abstract

The genus *Camelus* is an interesting model to study adaptive evolution in the mitochondrial genome, as the three extant Old World camel species inhabit hot and low-altitude as well as cold and high-altitude deserts. We sequenced 24 camel mitogenomes and combined them with three previously published sequences to study the role of natural selection under different environmental pressure, and to advance our understanding of the evolutionary history of the genus *Camelus*. We confirmed the heterogeneity of divergence across different components of the electron transport system. Lineage-specific analysis of mitochondrial protein evolution revealed a significant effect of purifying selection in the concatenated protein-coding genes in domestic Bactrian camels. The estimated *dN/dS* < 1 in the concatenated protein-coding genes suggested purifying selection as driving force for shaping mitogenome diversity in camels. Additional analyses of the functional divergence in amino acid changes between species-specific lineages indicated fixed substitutions in various genes, with radical effects on the physicochemical properties of the protein products. The evolutionary time estimates revealed a divergence between domestic and wild Bactrian camels around 1.1 [0.58–1.8] million years ago (mya). This has major implications for the conservation and management of the critically endangered wild species, *Camelus ferus*.

## Introduction

The genus *Camelus* is an interesting model to study the role of natural selection in the mitogenome as a driving force for adaptive divergence. The three extant *Camelus* species exist in very different habitats, each encompassing environmental pressures potentially resulting in the evolution of adaptations specific to each species. Dromedaries (*Camelus dromedarius*) inhabit semi-arid and hot desert regions of North and East Africa, Arabian Peninsula and southwest Asia^1, 2^, whereas domestic Bactrian camels (*Camelus bactrianus*) are distributed over central (Kazakhstan, Iran) and eastern (Russia, Mongolia, China) Asia, ranging from rocky mountains to flat and cold (semi-) deserts^3–5^. The wild two-humped camels (*Camelus ferus*) once were distributed over eastern and central Asia, adapted to arid and cold plains and hills^4, 6^. Today, this critically endangered wild species (IUCN 2014) can only be found in the Chinese Taklimakan and Lop Noor deserts and in the Mongolian Great Gobi Strictly Protected Area “A”, with a population census indicating as few as 2,000 individuals remain^7, 8^.

The ancestors of *Camelus* emerged on the North American continent 35–40 mya. After splitting into New World (Lamini) and Old World (Camelini) camels around 16.3 mya^9, 10^, the latter migrated via the Bering land bridge to the eastern hemisphere (the Old World), while the ancestors of llamas and alpacas spread to South America. Although the differentiation of the two domestic Old World camel species, dromedary and Bactrian camel, has long been established and estimated at around 4.4–8 mya^9, 10^, the wild two-humped camel (*C*. *ferus)* has been recognized only lately as a separate species^11–14^. However, a reliable estimate for the divergence time between domestic and wild Bactrian camels has been missing. Contrary to the last remaining wild relatives of the domestic Bactrian camel, the wild ancestors of the dromedary became extinct less than one millennium after the domestic form appeared (3,000–4,000 years ago), contributing to its domestication with multiple introgressions^15^.

Inferring the evolutionary and demographic history of wild and domestic species using mitochondrial DNA (mtDNA) has been established for a long time^16^. The relatively small mitogenome (16–17 kb) encodes a suite of 13 proteins, which interact with the nuclear DNA to build the oxidative phosphorylation (OXPHOS) pathway. Although these proteins are involved in key processes in the cells, the evolution of mtDNA has long been considered nearly neutral, and many studies have neglected the direct impact of positive and/or negative selection in shaping mitogenome diversity. A growing number of studies, however, favor the view that variation in the mitogenome is not selectively neutral (*e*.*g*., refs 17–21). Purifying selection has been implicated as a predominant force in shaping mitogenome evolution by limiting the accumulation of deleterious mutations at evolutionary constrained sites (*e*.*g*., cytochrome oxidase subunits (COX), NADH dehydrogenase subunits (ND), and cytochrome b (CYTB))^17, 22–25^.

Positive selection has been associated with shaping the mitogenome diversity in various species, such as snakes^26^, salmonids^27^, killer whales^28^, and across the mammalian phylogeny^29, 30^. Positive selection in the mitochondrial genes (*e*.*g*., COX, ATPase complexes, CYTB) has been shown to be involved in adaptation to different environmental pressures such as high altitude^31–33^ or temperature^18, 34–36^.

The most common approach in detecting selection in protein-coding genes involves estimating the rates of nonsynonymous (*dN*) and synonymous (*dS*) nucleotide substitutions and calculating the rates ratio as ω = *dN/dS*. In the McDonald-Kreitman (MK) test, the neutrality index (NI), or the excess of polymorphisms within species compared with the fixed substitutions between species, is used as an indicator to investigate the role of selection in shaping mitochondrial divergence between species^37^.

In the case of mtDNA, its high mutation rate can create intensive background noise hiding potential positively selected sites of adaptive value. Moreover, the commonly used methods such as *dN/dS* ratio and McDonald-Kreitman test are biased towards moderately conserved protein-coding genes^38^. The accurate diagnosis of individual amino acid changes with adaptive or non-adaptive effects is mathematically difficult because multiple nonsynonymous changes are required to satisfy the condition of *dN* > *dS* in a statistically significant manner^39^. One way to circumvent this problem is to analyze specific amino acid changes with adaptive values. Insights about selection acting on specific mitochondrial genes can be gained from branch-site tests on single codons^40^ and also by estimating lineage-specific deviations of *dN/dS* ratios in single genes^41^. Additional methods target the detection of positive selection at the mitochondrial level by comparing patterns of physicochemical changes of amino acids in the context of their effects on protein structure^42–44^. The technique of targeting changes in amino acid properties should be more sensitive than the *dN/dS* approach^45, 46^, and results in a high degree of resolution for the diagnosis of individual amino acid changes with adaptive values.

In this study, we screened for signatures of natural selection and divergence in 27 mitogenomes of the three different Old World camelid species. Specifically, we used a hierarchical approach and investigated (i) mitogenome evolution in the genus *Camelus* under a neutral model *versus* a model of natural selection; (ii) the evolution of mitochondrial protein-coding genes focusing on lineage-specific evolutionary changes; (iii) selection pressure on single sites in protein-coding genes within the *Camelus* lineage using a codon-based analysis; and (iv) changes in the physicochemical properties of functional proteins caused by amino acid replacements.

Finally, to gain knowledge about the evolutionary history of Old World camelids we estimated divergence times between domestic Bactrian camels and their wild, critically endangered relatives as well as between one- and two-humped camels using a Bayesian approach. Our study contributes to the understanding of evolutionary processes in Old World camels, and we provide support for the conservation and management of the critically endangered wild species *Camelus ferus*.

## Results

### Mitochondrial gene diversity and differentiation among Camelus

We analyzed 27 complete mitogenomes of Old World camels (Table S1), including three sequences from GenBank (*C*. *dromedarius*: NC_009849.1, *C*. *bactrianus:* NC_009628.2, *C*. *ferus*: NC_009629.2) to investigate signals of selection in *Camelus* possibly due to adaptation to different environmental regimes. With a mean read coverage of 20X [10–26X] after correcting for a maximal read coverage of 30, the alignment of all mitogenomes displayed a total length of 16,390 bp excluding repetitive regions and contained 1,347 variable sites (1,262 parsimony-informative, 85 singletons). We calculated the mitogenome diversity within each of the three Old World camel species and found higher haplotype (*H*_d_) and nucleotide diversity (π) in dromedaries (*H*_d_ = 1.0, π = 0.00169) compared to domestic (*H*_d_ = 0.952, π = 0.00132) and wild (*H*_d_ = 0.822, π = 0.00131) Bactrian camels, respectively (Table 1). Tajima’s D^47^ (−2.003, *P* < 0.01) as well as Fu and Li’s F^48^ (−2.580, *P* < 0.02) were significantly negative in dromedaries, whereas in wild camels they were positive (2.331, *P* < 0.05 and 1.772; *P* < 0.02, respectively). For the domestic Bactrian camels we could not reject the hypothesis of selective neutrality for Tajima’s D (−0.004, *P* > 0.10) and Fu and Li’s F (0.298, *P* > 0.10) (Table 1). The tests for past population growth based on the number of pairwise nucleotide differences considering a small sample size, *i*.*e*., Ramos-Onsins and Rozas R2 statistic^49^, as well as Fu’s Fs (−1.206, *P* < 0.002) resulted in a significant expansion signal only for dromedaries (Table 1).

**Table 1:** Mitogenome diversity in the three Old World camel species.

Species	n	Length^a^ (bp)	S	h	*H*_d_ (SD)	k	θ_π_ (SD)	θ_S_ (SD)	Tajima’s *D*	Fu and Li’s F test	Ramos-Onsins and Rozas (R2) statistic	Fu’s Fs statistic
*C*. *dromedarius*	10	16375	131	10	1.0 (0.045)	27.644	0.00169 (0.00097)	0.00283 (0.00116)	−2.00336**	−2,57981**	0,2330 ns	−1,206**
*C*. *bactrianus*	7	16385	53	6	0.952 (0.096)	21.619	0.00132 (0.00028)	0.00132 (0.00061)	−0.00364 ns	0.29791 ns	0,1555 ns	1,466 ns
*C*. *ferus*	10	16383	41	5	0.822 (0.097)	21.444	0.00131 (0.00017)	0.00088 (0.00038)	2.33046*	1.77232**	0,2547 ns	6,667 ns

^a^Alignment excluding repetitive parts of the CR, gaps and missing data; n = number of samples; S = segregating sites; h = number of haplotypes; *H*_d  _ = haplotype diversity; k = average number of nucleotide differences; θ_π_ = theta estimator based on the mean number of nucleotide differences; θ_S_ = Waterson’s theta estimator based on the number of segregating sites; SD = standard deviation values. Statistical significance: *P < 0.05; **P < 0.02, ns ﻿= not significant.

The average number of nucleotide differences between species (n) and the net nucleotide substitutions per site (D_a_) between the pairwise species comparisons were calculated as n = 520.9/588.9 and D_a_ = 0.069/0.070 (dromedary *vs*. domestic/wild Bactrian camel) and n = 160.7 and D_a_ = 0.018 (domestic *vs*. wild Bactrian camel) (Tables S2–4). We observed substantial differentiation in protein-coding genes between the pairwise comparisons of Old World camels (dromedary *vs*. Bactrian camel, dromedary *vs*. wild camel and Bactrian *vs*. wild camel). This resulted in 862, 874 and 223 fixed synonymous and nonsynonymous substitutions (D_s_ + D_n_), leading to a permanent replacement (D_n_) of 84, 90 and 25 amino acids, respectively (Tables S2–4). In all pairwise comparisons, net divergence (D_a_) varied substantially between genes; however, it was lower in the two rRNA subunits (12S, 16S) and in the concatenated tRNAs in comparison with protein-coding genes (Tables S2–4).

To investigate the heterogeneity of divergence across gene classes with different functions we examined the divergence level in protein-coding genes classified into different mitochondrial components of the electron transport system (ETS) complexes. In each pairwise comparison among the three species, we observed the highest differentiation in ETS III (D_a (db)_ = 0.092, D_a (df)_ = 0.099, D_a (bf)_ = 0.023) and the lowest in ETS IV (D_a (db)_ = 0.068, D_a (df)_ = 0.069, D_a (bf)_ = 0.017). The D_a (bf)_ between domestic and wild Bactrian camels showed low differentiation among all ETS complexes (Da _ETS I_ = 0.022, D_a ETS III_ = 0.023, D_a ETS IV_ = 0.017, D_a ETS V_ = 0.018) (Table S5).

### Signals of purifying selection in the genus Camelus

#### Mitogenome protein evolution under a neutral model versus a model of natural selection

The global MK test^37^ performed pairwise on the concatenated protein-coding genes (11,379 bp) in the three Old World camel species showed an excess of amino acid polymorphism in dromedary and domestic Bactrian camels (P_n_/P_s_ = 0.21) as well as a deficiency of interspecific fixed amino acid replacements (D_n_/D_s_ = 0.10). This resulted in a NI of 2.0 (*P* = 0.006) for dromedary and domestic Bactrian camels, indicative of purifying selection. A similar NI of 2.17 (*P* = 0.035) was detected in the pairwise comparison between domestic and wild Bactrian camels. In contrast, between dromedary and wild Bactrian camels we could not identify any signal of positive or negative selection (NI = 1.41, *P* = 0.244). Moreover, we compared the one- and two-humped camels, resulting in NI = 1.46 (*P* = 0.041) (Table S6).

Next, we investigated lineage-specific mtDNA protein evolution by performing the “extended MK test”^50^ for both concatenated protein-coding genes as well as each gene separately. In dromedaries, the NI indicated an excess of nucleotide polymorphism in ND6 in respect to its divergence from the domestic (NI = 13.33, *P* = 0.009) and wild Bactrian camels (NI = 14.66, *P* = 0.007), suggesting purifying selection against mildly deleterious mutations in this gene (Tables S7 and 8). In domestic Bactrian camels, we detected significant effects of purifying selection in concatenated protein-coding genes with respect to dromedaries (NI = 3.47, *P* = 0.001) and wild Bactrian camels (NI = 2.97, *P* = 0.0083) (Tables S9 and 10). The excess of replacement polymorphism was not significant in the wild Bactrian camel lineage (Tables S11 and 12). The significant results of the MK test (global and extended) indicating signals of purifying selection in the mitogenomes of the genus *Camelus* are summarized in Table 2.

**Table 2:** Summary of the significant results of the global and extended MK tests indicating purifying selection in *Camelus* mitogenomes.

	Selection test	mtDNA gene	NI (*P*)
dromedary *vs*. domestic Bactrian camel	g-MKT	PC genes	2 (0.006**)
	e-MKT	ND6	13.33 (0.009**)
dromedary *vs*. wild Bactrian camel	e-MKT	ND6	14.66 (0.007**)
domestic Bactrian camel *vs*. dromedary	e-MKT	PC genes	3.47 (0.001**)
domestic *vs*. wild Bactrian camel	e-MKT	PC genes	2.17 (0.035*)
domestic *vs*. wild Bactrian camel	e-MKT	PC genes	2.97 (0.008**)
one- *vs*. two-humped camels	g-MKT	PC genes	1.48 (0.041*)

The two-tailed *P*-values of Fisher’s exact tests are shown. *P*-values with asterisk are significant with the following significant levels *P < 0.05; **P < 0.01. PC = concatenated protein coding genes, g-MKT = global MKT, e-MKT = extended MKT, NI = neutrality index.

#### Evolution of mitochondrial protein-coding genes focusing on the lineage-specific evolutionary changes

To test for selective pressure acting on a particular *Camelus* lineage, we used a maximum likelihood (ML) approach and examined variation in the ratio of *dN/dS* (ω) on the codon-based alignments of ten different mammalian species including Old World camels (*C*. *dromedarius*, *C*. *bactrianus*, *C*. *ferus*), New World camels (*Lama glama*, *L*. *guanicoe*, *Vicugna pacos*, *V*. *vicugna*), *Bos taurus*, *Pantholops hodgsonii* and *Homo sapiens* (see Table S1 for accession numbers). In the background model, the uniform *dN/dS* (ω) ratio across the reconstructed phylogeny (HKY + G + I) of concatenated protein-coding genes was estimated at ω = 0.035. In the species-specific model, which was a significant improvement over the background model (likelihood ratio test [LRT], χ^2^ = 179.7, df = 64, *P* < 0.001), the highest *dN/dS* ratio (ω = 0.063) was estimated in wild camels followed by dromedaries (ω = 0.049) and domestic Bactrian camels (ω = 0.041) (Fig. 2). The two-humped camel lineage displayed an ω of 0.025 preceding its separation into the domestic and wild Bactrian camels.

**Figure 1:**
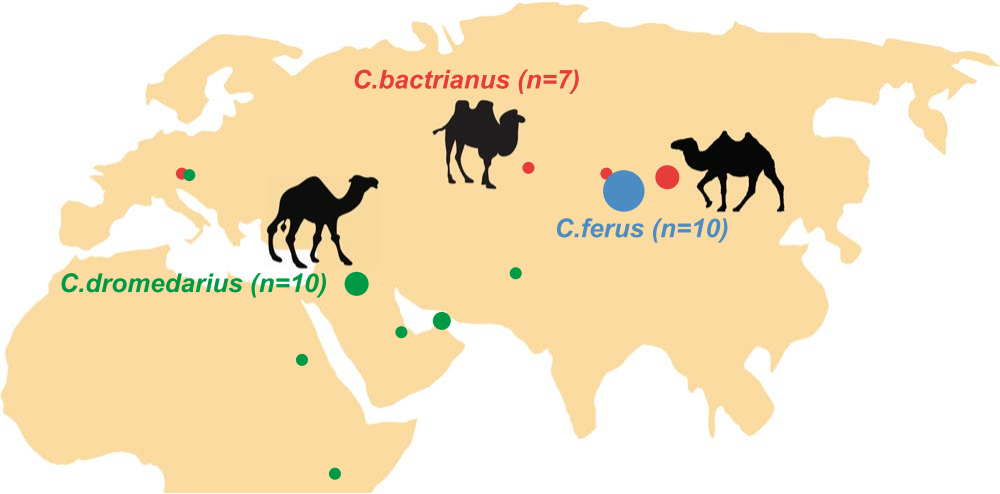
Geographical locations of the samples used in this study, including three published mitogenomes obtained from GenBank. The size of each circle is proportional to the sample size for that location. Colors correspond to the three Old World camel species, and the number of individuals per species are shown within parentheses. Detailed sample information is given in Table S1. The map and figures are adopted from https://openclipart.org and are licensed under Creative Commons Zero 1.0 License.

**Figure 2:**
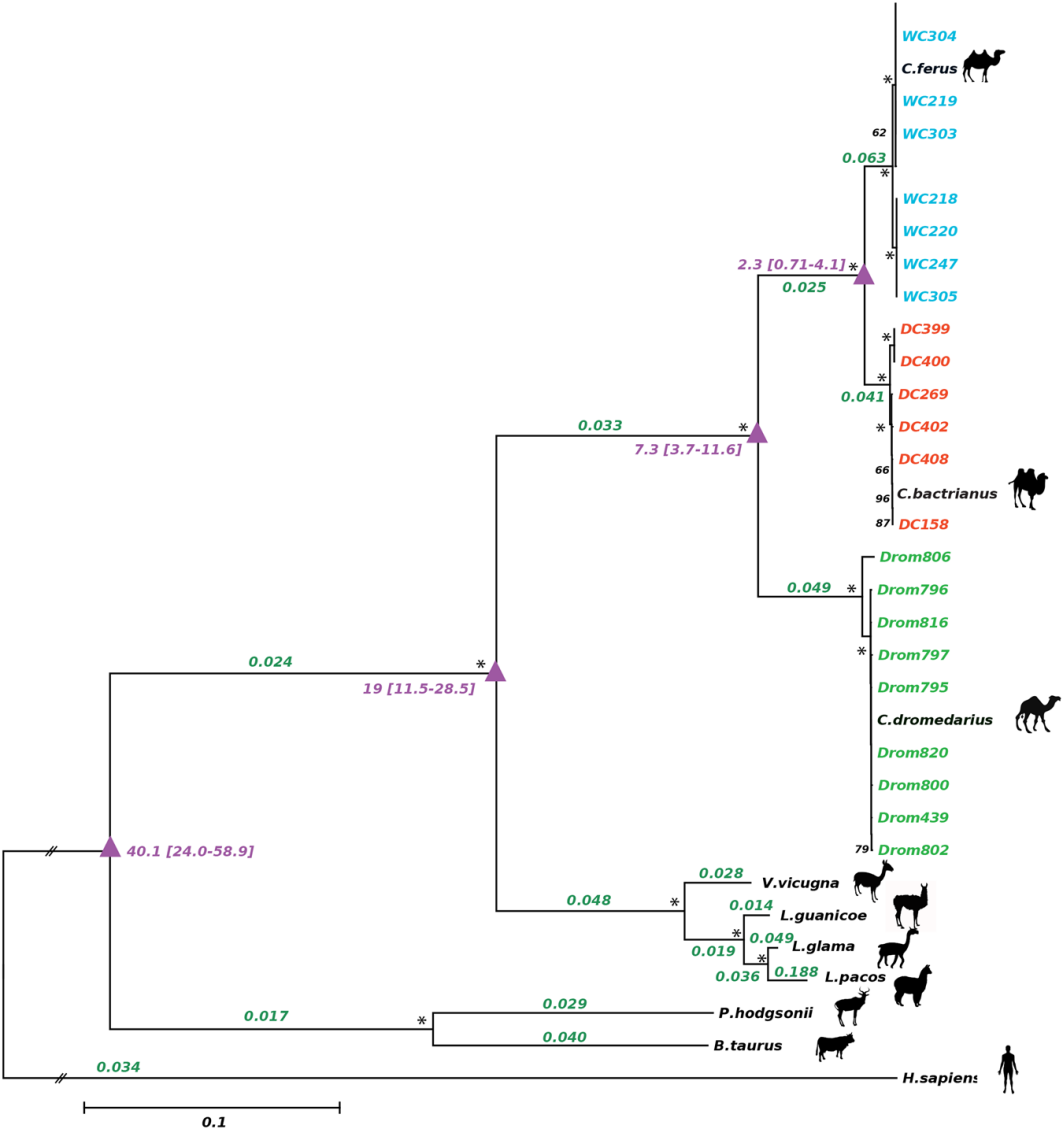
The Maximum likelihood phylogenetic tree inferred from 11,379 bp concatenated protein-coding genes from 10 different species. The HKY substitution model with a gamma distribution and proportion of invariant sites (+GI) was used to model evolutionary rate differences. Lineage-specific *dN/dS* values for concatenated protein-coding genes are indicated in green over each branch. The estimated divergence time (mya; using first and second protein-coding positions) of each evolutionary lineage is shown in purple color, with confidence intervals in brackets. The statistical support for each node is based on 100 bootstrap replicates, with values (in black) on nodes indicating bootstrap supports above 50%; an asterisk indicates a value of 100%. Branch lengths are scaled as the number of base pair changes per site, with the exception of the human outgroup sequence which is rescaled for aesthetic purposes. The figures depicted on the phylogenetic tree are adopted from https://openclipart.org, and are licensed under Creative Commons Zero 1.0 License.

#### Selection pressure on single sites in protein-coding genes within the Camelus lineage using a codon-based analysis

Using the branch-site model A (foreground lineage *vs*. background lineages) implemented in CODEML (PAML)^51^, the Bayes empirical Bayes (BEB) analysis indicated a total of 18 sites under positive selection in the entire *Camelus* lineage (Table S13). We detected 4 sites (in ND2, ATP6), 10 sites (in ND1, ND2, COX3, ND3, ND4, ND5) and 12 sites (in ND2, ATP8, ND4, ND5, CYTB) under positive selection in dromedaries, Bactrian camels and wild camels, respectively (Table S13). However, the significance of the detected sites could not be confirmed in the subsequent LRTs (Table S13).

#### Changes in the physicochemical properties of functional proteins by amino acid replacements

The analysis of functional divergence in amino acid changes was performed using TreeSAAP software^52^, which takes into account the magnitude of the impact of the amino acid replacement on local physicochemical properties of the protein. Changes with a magnitude ≥6 and *P* < 0.001 were considered as indicating positive directional selection for a given physiochemical property^52^. From a total of 84 (dromedary *vs*. Bactrian camel), 90 (dromedary *vs*. wild camel) and 25 (domestic *vs*. wild Bactrian camel) fixed nonsynonymous substitutions between *Camelus* species, we found evidence of positively selected sites in ATP8 (1 site), ATP6 (2 sites), ND3 (2 sites), ND5 (15 sites) and ND6 genes (4 sites) (Table S14). In these protein-coding genes, we observed 12, 14 and 12 ancestral amino acids in dromedary, domestic and wild Bactrian camels, respectively (Table S14). These amino acid changes affect physicochemical properties such as alpha-helical tendencies (*P*_α_), equilibrium constant (ionization of COOH) (pK′), polar requirement (*P*_*r*_), surrounding hydrophobicity (*H*_*p*_), power to be at the middle of alpha-helix (α_m_), isoelectric point (*pHi)*, and solvent accessible reduction ratio (R_a_).

### Divergence time estimations among Camelidae

We used a Bayesian approach to estimate the 95% credibility intervals (CIs) of divergence times between the camelid species (Fig. 2 and Table 3). We performed five independent runs of MULTIDIVTIME^53^, all of which indicated good convergence (all corrected scale reduction factors = 1.0). Furthermore, the multivariate potential scale reduction factor (MPSRF)^54, 55^ was 1.001 and 1.01 for analyses including all sites and only first and second positions, respectively, supporting that the results across all runs had reached a stationary distribution. The mean and 95% CI for the divergence between Camelidae and Bovidae was estimated at 41.4 [24.7–60.6] mya. Within the Camelidae, the mean divergence time between the Camelini and Lamini tribes was 15.8 [CI 9.2–23.2] mya. The mean divergence between one-humped and two-humped camels occurred around 5.3 [CI 2.9–7.7] mya, and domestic and wild Bactrian camels split at about 1.1 [CI 0.58–1.8] mya. The estimates for divergence times when restricted to only the first and second codon positions were similar, albeit slightly higher than estimates from all sites (Table 3). For example, this analysis suggested the divergence between domestic and wild Bactrian camels had occurred 2.3 [CI 0.71–4.1] mya.

**Table 3:** Divergence time estimates among Camelidae.

Phylogenetic node	All sites	First and second protein-coding positions only
Camelidae & Bovidae	41.4 [24.7–60.6]	40.1 [24.0–58.9]
Camelini & Lamini	15.8 [9.2–23.2]	19 [11.5–28.5]
*C*. *dromedarius* & [*C*. *bactrianus* + *C*. *ferus*]	5.3 [2.9–7.7]	7.3 [3.7–11.6]
*C*. *bactrianus* & *C*. *ferus*	1.1 [0.58–1.8]	2.3 [0.71–4.1]

Mean and 95% posterior credibility intervals (within brackets) of divergence time estimates for the four nodes of interest produced using MULTIDIVTIME. All values are given in millions of years ago (mya).

The mtDNA saturation analysis showed that the %Ti did not approach 50% until a sequence divergence of around 0.23 and 0.12 for all sites and first and second positions, respectively (Figure S1). The mean sequence divergence across all genes for Bovidae *vs*. Camelidae pairwise comparisons was 0.23 and 0.10 for all sites and first and second positions, respectively. This suggests that the Bovidae-Camelidae divergence time may be underestimated due to saturation, whereas shallower nodes are less likely to be affected.

## Discussion

We investigated the evolutionary history of Old World camelids and studied the role of natural selection in shaping mitochondrial diversity. The three species are well-adapted to different environmental regimes as their habitats are spread over different altitudes and temperatures. Particularly, we were interested in estimating the divergence time between the domestic and wild Bactrian camel because a reliable estimate had not been obtained previously.

### Diversity and differentiation in the mitochondrial genes among Old World camels

We observed higher haplotype and nucleotide diversity in the ten investigated dromedary mitogenomes compared to the seven domestic and ten wild Bactrian camels (Table 1), respectively. The geographically larger distribution of the dromedary samples over three continents and seven countries *versus* the domestic Bactrian camel specimen collected from Mongolia, Kazakhstan and Austria (with potential Mongolian origin) in principle could account for these differences. Similarly, the wild two-humped camel samples originated from one national park, the Strictly Protected Area “A” in the Mongolian Gobi desert (Table S1), a consequence of their small census size and limited geographic habitat in Mongolia and China.

During the process of domestication, population growth and dispersion of animals across a wider geographic range can be captured from molecular signals of sudden expansion^56^. In this study we obtained significant negative values of Tajima’s *D*, and Fu and Li’s F test in dromedaries (Table 1), which can indicate demographic expansion and/or positive selection. Evidence for recent population expansion in the context of dromedary domestication was confirmed by partial mitochondrial ND5, CYTB and CR analysis from over 600 individuals throughout their range^15^. On the contrary, the positive values of Tajima’s D and Fu and Li’s F test in the wild two-humped camels are indicative of a decrease in population size and/or balancing selection. These results are in agreement with a reduced genetic variation and small effective population size observed in wild Bactrian camels^7, 57^.

Overall, we calculated a mitogenome differentiation of 6.9% and 7% between dromedaries and domestic and wild Bactrian camels (Tables S2 and 3), respectively; whereas the sequence differences among two-humped camels showed 1.8% (Table S4). This corresponds to previous estimates (1.9%–2%) on partial mitochondrial genome sequences including ND5, CYTB and CR^13, 14^.

Investigating the heterogeneity of divergence across the different gene classes the rRNAs (12 S and 16S) and tRNAs, which are required for mitochondrial protein synthesis, were highly conserved. This is in agreement with general slow divergence rates of mitochondrial RNA genes observed in other mammals^58–62^. The observed rates of molecular evolution in different protein-coding genes of the ETS complexes reflected the differences in their functional constraints. In the ETS complexes the rates of molecular evolution followed the order CYTB > NDs > ATPs > COXs, with the majority of fixed differences observed in CYTB and the fewest in the COX genes. Functional modifications in the CYTB protein may be involved in physiological adaptation to different thermal environments^25, 30, 36^. The high variation observed in CYTB in the *Camelus* lineage could thus be explained with more specialized metabolic requirements in camels, like the adaptation to low-energy diet or living at higher altitudes in the case of Bactrian camels. A pattern of high variation in CYTB was also observed in other species with specialized metabolic requirements, such as the elephant, dugong, sloth and pangolin^29^. Higher rates of evolution in ND genes compared to the ATP and COX were reported previously in different species of insects, fish and mammals^63, 64^. As reported in other studies, nonsynonymous mutations in the ND genes are generally less deleterious than mutations in the COX genes, which oftentimes greatly alter functional properties of the protein and fitness^24, 31, 65, 66^. When we took into account the different sequence lengths of the ETS complexes, we observed the highest number of fixed nonsynonymous substitutions in the ATP genes in pairwise comparison between dromedary *versus* domestic and wild Bactrian. In all comparisons, the COX genes contained the lowest number of nonsynonymous substitutions, indicating their high evolutionary constraint in the *Camelus* lineage.

### Signatures of purifying selection in the mitogenomes of Camelus

#### Mitogenome protein evolution under a model of natural selection

The pattern of intraspecific polymorphism in dromedary and interspecific divergence to domestic/wild Bactrian camel indicated the presence of purifying selection in the ND6 gene. This result showed that selection prevents the accumulation of slightly deleterious mutations in ND6, which is suggested to be one of the active subunits of the enzyme involved in OXPHOS pathways^67^. No evidence of selection was found in other protein-coding genes in dromedaries, probably a result of insensitivity of the extended MK test for the restricted number of polymorphisms and divergence in species with recent population expansion^34^. In domestic Bactrian camels, we did not detect signals of purifying selection in any particular gene; however, we observed a significant level of purifying selection over the concatenated protein-coding regions.

#### Evolution of mitochondrial protein-coding genes focusing on the lineage-specific evolutionary changes

The low *dN/dS* ratio estimated for the *Camelus* lineage (*dN/dS* = 0.033) indicated the effect of purifying selection on the mitochondrial protein-coding genes in this lineage. Purifying selection has been shown to be the main driver in shaping mitogenome diversity^17, 22–25^. However, it has been observed that domesticated species have a higher *dN/dS* ratio than their wild counterparts; a result possibly attributed to relaxed purifying selection during domestication (*e*.*g*., yak^68^, goat^69^). We observed this pattern in South American camelids, but contrary to this hypothesis, in wild two-humped camels we estimated slightly higher *dN/dS* ratio (*dN/dS* = 0.063) in comparison to the two domestic species (dromedaries *dN/dS* = 0.049; Bactrian camels *dN/dS* = 0.041). Wild camels are a sister species to the domestic Bactrian camels and have never been domesticated. Their original population size has been reduced over 80% during the last century and ﻿probably numbers less than 2,000 individuals^7, 8^. The current extremely small population size might be a reason for the relaxed pressure of purifying selection acting on the mitochondrial protein-coding genes, which could be explained by the stronger effect of genetic drift than natural selection in populations with smaller size^70–73^. Furthermore, compared with other domesticated ungulates, domesticated camels (dromedaries and Bactrian camels) have maintained unusually high levels of genetic variation and lack the extensive secondary bottlenecks experienced during breed formation^15^. The combination of the small population size in wild camels and the chance of more effective purifying selection in the domesticated species could be responsible for this result. Nonetheless, we conclude that purifying selection acts to maintain the stability and efficiency of the OXPHOS system, which are the requirements for aerobic cell respiration and energy production.

#### Selection pressure on single sites in protein-coding genes within Camelus

Despite the low *dN/dS* ratio values across the examined *Camelus* phylogeny indicating purifying selection, the BEB analysis of the branch-site model A recovered 18 codons potentially under positive selection (BEB > 50%, including one codon BEB > 95%) in seven genes (Table S13). However, their significance could not be further confirmed with the LRT. The BEB approach is not a direct estimator of positive selection and is used to allow sampling error in parameter estimates. Considering the small sample size and possibly large parameter estimate errors, there is very little power to detect individual sites under selection. Similarly, Matosiuk *et al*.^58^ identified 12 sites in roe deer lineage that may have been positively selected, which their significance could not be confirmed with subsequent LRT.

#### Changes in the physicochemical amino acid properties of functional proteins

We detected candidate positions for positive selection at fixed nonsynonymous substitutions in ATP8 (1 site), ATP6 (2 sites), ND3 (2 sites), ND5 (15 sites) and ND6 genes (4 sites) when comparing species-specific lineages (Table S14). The observed amino acid changes can affect physicochemical properties such as alpha-helical tendencies, equilibrium constant (ionization of COOH), polar requirement, surrounding hydrophobicity, power to be at the middle of alpha-helix, isoelectric point, and solvent accessible reduction ratio. The ancestral amino acid states are equally represented in dromedary, domestic and wild Bactrian camels (Table S14).

The detection of positive selection by comparing physicochemical changes of amino acids is very sensitive, as in highly conserved genes even single amino acid changes can be adaptive if they cause biochemically advantageous characterization. However, a single base change seems too weak to be identified as a positively selected site with the *dN/dS* ratio approach^45, 46, 74, 75^. As a reaction to this potential weakness in *dN/dS*, we applied a physicochemical property model in TreeSAAP and characterized positive destabilizing selection that correlated with seven biochemical functional shifts in local regions of the proteins. McClellan *et al*. (2005) argued that when this radical amino acid changes are favored by selection, they potentially result in local directional shifts in biochemical function, structure, or both, representing the unambiguous signature of molecular adaptation^46^. The observed changes in the amino acid properties can affect protein functions in different ways. First, we identified an increase or decrease in the alpha-helical tendency (*P*_α_) in the ND5 gene caused by five or three amino acid changes, respectively. Increasing the alpha-helical tendencies results in a longer, more rigid alpha helix, which makes interactions with other amino acid motifs more difficult. On the contrary, decreasing this property allows for a more flexible and open alpha helix, which increases the accessibility at protein interfaces^76^. Second, we detected an increase (eight amino acid changes) or decrease (three changes) in the equilibrium constant for the ionization of COOH (pK′) in ND6 and ND5, respectively, which make the region more or less water-soluble and hydrophilic. An increase in hydrophilicity is very important regarding a reduced reactive oxygen species (ROS) production and increased longevity during calorie restriction^77^. An increase in surrounding hydrophobicity (*H*_*p*_) property in ND3 gene, indicated that the regions surrounding these amino acid sites become less hydrophilic and more hydrophobic by introducing an isoleucine instead of a threonine.

### Long-term divergence between domestic and wild Bactrian camels

Until recently, the critically endangered wild two-humped camels have been either considered to be feral (domestic runaways; reviewed in Peters & van den Driesch^78^), or classified as a subspecies of the domestic Bactrian camel, *Camelus bactrianus ferus* ^12^. However, earlier studies on partial mitochondrial genes indicated a high amount of differentiation (1.8–2%; refs 13, 14 and 79) between domestic and wild two-humped camels, and the International Commission of Nomenclature^80^ designated the species status “*Camelus ferus”* to the wild camel^81^. Genome-wide comparison between the two species confirmed their differentiation^12^, however, no divergence time was investigated. Here, we estimated the split between domestic Bactrian camels and their wild relatives to 1.1 [0.58–1.8] mya u﻿sing all sites,﻿ and to 2.3 [﻿0﻿.71–4.1] mya using first and second protein-coding positions only (Table 3); long before domestication started around 4000–6000 ya^82^. With no mitochondrial haplotype sharing and a high number of fixed nucleotide polymorphisms (n = 233) between them, we conclude that wild camels are not only a separate species but also not the direct ancestors of contemporary domestic Bactrian camels. Our modern livestock consequently was domesticated from different ancestral populations, which had diverged from their wild sister species around 1.1–2.3 mya, and became extinct after founding the modern domestic Bactrian camel gene pool. A similar evolutionary history can be observed in the Przewalski and the domestic horse^83, 84^. Finally, our divergence time estimates between Camelidae and Bovidae 41.4 [24.7–60.6] mya as well as between Camelini and Lamini 15.8 [9.2–23.2] mya were in line with the latest genome-wide comparisons between the different species (42.7 [33.2–51.0] and 16.3 [9.4–25.3], respectively)^9^. Considering that most wild relatives of our domesticates (including llama and alpaca) are still alive, our results emphasize the evolutionary importance of *Camelus ferus* as the last wild representatives of the Old World camelids.

## Methods

### Sampling and sequencing of complete mtDNA

Genomic DNA was extract ed from whole blood of 24 specimens of Old World camelids (Table S1 and Fig. 1), using Master Pure^TM^ DNA purification kit for blood (Epicentre version III). This sampling was designed to span the known phylogenetic tree of Old World camelids, and to include mtDNA of species that are well adapted to hot, arid (nine dromedaries) and cold, dry (nine wild and six domestic Bactrian camels) environments. Blood samples were retrieved commensally either during routine veterinary treatment or during a radio-collaring project for Mongolian wild camels. All data sets were collected within the frames of the legal requirements of Austria and Mongolia. Capture and collaring of wild camels was conducted within a cooperation agreement between the International Takhi Group and the Mongolian Ministry of Nature, Environment and Tourism (signed on 15.02.2001 and renewed on 27.01.2011). The 500 bp paired-end Illumina library preparation and sequencing of each sample on a single lane of Illumina HiSeq 2000 platform was performed within the framework of a whole-genome re-sequencing study of Old World camelids (Fitak *et al*. in preparation). Three reference mitogenomes were retrieved from GenBank (Accession numbers NC_009849.1, NC_009628.2, NC_009629.2).

### Read processing and alignments

The raw sequence reads for each individual were trimmed at the 3′ end to a minimum phred-scaled base quality of 20 and minimum length of 50 bp (base pairs) using POPOOLATION v1.2.2^85^. For each species, the trimmed reads were mapped against the corresponding mitochondrial reference genome (*C*. *dromedarius* Genbank Accession no: NC_009849.1, *C*. *bactrianus:* NC_009628.2, and *C*. *ferus*: NC_009629.2), using BWA v0.7^86^ with the following parameters (-l 200 -n 0.01 -o 1 -e 12 -d 12 -t 6). Duplicate reads were removed using Picard-tools ‘MarkDuplicates’ v.1.89 (http://www.picard.sourceforge.net) and only the filtered reads, which were properly paired and unambiguously mapped with mapping quality score >20 were used for further analyses. The consensus sequence for each sample was generated using CLC Genomics Workbench 6.5.1 (http://www.clcbio.com). The consensus mitogenomes were aligned with CodonCode Aligner v.3.7.1 (Codon Code Corporation, USA). The mitochondrial sequences of all 13 protein-coding genes, two rRNA subunits (12S, 16S) and control region of the three camel species were identified using the corresponding reference sequences of dromedary, domestic and wild Bactrian camel. The overlapping fragments of all sequences were examined to ensure complete sequence coverage. To avoid nuclear insertions of mitochondrial sequences (Numts), all amino acid sequences of protein-coding genes were screened for frameshift mutations and/or early stop codons. As additional quality control we visually screened the sequence reads for misalignments and/or large gaps using Integrative Genomics Viewer v.2.3^87^.

### Mitochondrial gene diversity and differentiation among Camelus

Summary statistics of the 27 camel mitogenomes, within and between species were calculated with DnaSP v5.10.1^88^. Within species diversity parameters included the number of polymorphic (segregating) sites (S), number of haplotypes (h), haplotype (*H*_d_) and nucleotide (π) diversity, average number of nucleotide differences (k) and Watterson’s theta (θ_S_) based on the number of segregating sites (Table 1). The software was also used to carry out neutrality tests including Tajima’s D^47^, Fu and Li’s F^48^, Fu’s Fs^89^, and Ramos-Onsins and Rozas R_2_ statistic^49^. Both D and F can detect historical population expansions and contractions, whereas Fs and R_2_ explicitly test past population expansion and are more powerful^49^. When testing for past expansions, R_2_ is preferred for small sample sizes (n ≤ 20) and Fs for larger sample sizes^49^. Significance and 95% confidence intervals of the Fs and R_2_ statistics were calculated with 1000 coalescent simulations. Pairwise differentiation comparisons between species comprised the average number of nucleotide differences between species (n), number of fixed synonymous (D_s_) and nonsynonymous substitutions (D_n_) between species, and a sliding window analysis (window size 100 bp, step size 25 bp) of the number of net nucleotide substitutions per site (D_a_) between species-specific lineages. The summary statistics of differentiation in pairwise comparisons between species were calculated for the protein-coding genes, rRNAs, tRNAs and the control region separately, with the overall differentiation as a sum of individual gene divergence.

### Mitochondrial genome evolution and screening for natural selection

To address our main question, whether mitogenomes in the genus *Camelus* evolve under a model of neutrality or natural selection, we employed four different approaches (*i*–*iv*): first, we performed a global MK test^37^ on the concatenated protein-coding genes (11,379 bp), using DnaSP v5.10.1^88^. We calculated the neutrality index (NI = (P_n_/P_s_)/(D_n_/D_s_); polymorphic sites/ fixed sites among species) at non-synonymous (*dN*) and synonymous (*dS*) sites in pairwise comparison between the three species. The significance of the NI values was assessed with the two-tailed Fisher’s exact test. Next, to explicitly examine which *Camelus* lineage had either a deficiency or excess of amino acid polymorphisms and as such might be affected by natural selection, we performed an extended MK test^50^ on the concatenated protein-coding genes, and each of 13 protein-coding genes separately. In the extended MK test we calculated polymorphisms within one species and the divergence (fixed SNPs) at nonsynonymous and synonymous sites in pairwise comparison.

In our second approach, we examined whether mitochondrial protein-coding genes in the *Camelus* lineage show any specific patterns of evolutionary rates. We assessed the variation in the ratio of *dN* to *dS* among the branches of a phylogenetic maximum likelihood (ML) tree using MEGA6^90^, with the best-fitting model HKY + G + I according to the Bayesian Information Criterion (BIC). Using the ML method implemented in the CODEML program of PAML v.4.8^51^, we applied the background and the branch-specific model on the 11,379 bp concatenated alignment (including gaps) of the mitochondrial protein-coding genes from ten different species (34 individuals). We included Old World (*C*. *dromedarius*, *C*. *bactrianus*, *C*. *ferus*) and New World camels (*Lama glama*, *L*. *guanicoe*, *Vicugna pacos*, *V*. *vicugna*), *Bos taurus*, *Pantholops hodgsonii* and *Homo sapiens*; all accession numbers are given in Table S1. While in the background model a single value of the *dN/dS* ratio (ω) is considered among all sites and branches, the branch-specific model allows a varying ω among the branches in the phylogeny^91^. We used a LRT to determine whether or not the branch-specific model was a significant improvement over the background model.

Third, we tested for selection in single sites of the protein-coding genes within the genus *Camelus* using the branch-site model A^40^ in CODEML. In this model, the ω value is allowed to vary among different branches as well as in the nucleotide sites of a particular branch (foreground lineage). We tested a null model (H0), in which the foreground branch (*Camelus sp*.) may have different proportions of sites under neutral selection than the background (*i*.*e*., relaxed purifying selection), and an alternative model (H1), in which the foreground branch may have a proportion of sites under positive selection. If in the alternative model a site was identified as potentially fixed due to positive selection (*dN/dS* > 1), its posterior probability was estimated with the Bayes empirical Bayes (BEB) approach^51^. For each model we retrieved the log likelihood values from which we computed the likelihood ratio tests (LRT) for the significance (*P-*value) of the sites detected under positive selection.

Finally, we investigated the effect of amino acid changes on the protein function, using TreeSAAP^52^. This program uses statistical models of molecular evolution and phylogenetic trees to predict positive selection based on amino acid physiochemical properties. TreeSAAP takes into account the magnitude of the impact of the amino acid replacements on local physiochemical properties and determines whether the observed degree of amino acid changes deviates from the neutral expectation. Radical magnitudes of changes ≥6, with *P* ≤ 0.001, are considered as an evidence for positive directional selection for a given physiochemical property^92^. To strengthen the accuracy and avoid false positive results we only used amino acid properties with the accuracy of detecting selection higher than 85% (i.e., 11 out of 31 sites were excluded) as recommended in^92^. In case of detecting fixed amino acid changes with effects on the physiochemical properties of a protein, we determined the ancestral state of the amino acid preceding the split of one- and two-humped camels, and domestic and wild Bactrian camels with the ML analysis in MEGA6^90^. We constructed the phylogenetic tree for each protein coding gene in eight different species (Camelini, Lamini, and *B*.*taurus*) in MEGA as described above and used these trees as input into TreeSAAP. We restricted the sites analyzed to only the fixed nonsynonymous substitutions in pairwise comparisons in the three camel species. This analysis allowed us to investigate whether the domestic species have more amino acid changes with a predicted negative or positive impact on the respective protein due to artificial selection during domestication.

### Divergence time estimation

We estimated the divergence times among *Camelidae* with MULTIDIVTIME^53^, which calculates posterior credibility intervals (CIs) of divergence times using a Bayesian approach and assuming a relaxed molecular clock with autocorrelation among rates. This method has been shown to produce posterior CIs that contain the true divergence time of simulated datasets ≥95% of the time as long as the assumption of autocorrelation among rates was not violated^93^. We followed the protocol outlined by Rutschmann^94^ for the 13 protein-coding DNA sequences. The analysis can be summarized as follows: for each gene’s alignment we determined the best-fit nucleotide substitution model and generated a ML tree topology in MEGA6 according to the procedure above. We calculated model parameters, including nucleotide frequencies, the transition/transversion rate ratio, and rate heterogeneity among sites for each gene using ‘baseml’ in PAML v4.8^51^. Next, branch lengths and a variance-covariance matrix were estimated using the ‘estbranches’ function in MULTIDIVTIME against the ML tree topology for the 10-species dataset described above. We then ran five independent chains of MULTIDIVTIME to estimate divergence times, with each chain containing 10^5^ burn-in steps followed by 10^6^ steps sampled every 100^th^ step (10^4^ samples in total). We parameterized the MULTIDIVTIME runs using rttm = 6.5, rtrate = 0.07, and brownmean = 0.3 and with equivalent standard deviations, respectively. The time between the ingroup root and median of the tips (rttm) corresponded to 1 unit ≈ 10 million years, and was retrieved from the TimeTree database^95^ as the mean time of the most recent common ancestor between the families Bovidae and Camelidae (65 mya). The rate (rtrate) was calculated as rttm divided by the median branch length between the ingroup root and tips. We set brownmean to approximately 2/rttm. We included a prior to constrain the node connecting the Camelini and Lamini tribes to 8–30 mya based upon both fossil^96, 97^, and molecular^98, 99^ evidence.

Prior to estimating posterior probabilities, we assessed convergence of the independent MULTIDIVTIME chains using the R package ‘boa’ v1.1.8^100^ to ensure the runs reached a stationary distribution. We examined each chain and its parameters (divergence time and rate for each node) independently for convergence using the corrected scale reduction factors (CSRF) from^54, 55^. A chain is suggested to have converged when the CSRF is 1, or more specifically, when the 97.5% upper confidence limit of the estimate is ≤1.2^54^. Furthermore, we also examined for convergence across all parameters and chains simultaneously using the multivariate potential scale reduction factor (MPSRF)^54, 55^. The MPSRF converges to 1 upon reaching stationarity when the number of steps is reasonably large. After convergence was verified, all chains were combined and the posterior density mean and 95% CIs were estimated for the parameters. We repeated the above analysis in MULTDIVTIME using only the first and second codon positions with a modified rtrate = 0.012.

Because mtDNA evolves rapidly, it is often subject to the effects of saturation (multiple mutations at a single site) – especially at deep divergence times^101, 102^. As a result, divergence estimates need to take into account the possible effects of saturation. To determine the potential for saturation effects in our dataset, we used the DNA saturation analysis suggested in ref. 101. Briefly, at low levels of divergence the ratio of transitions to transversions (Ti/Tv) in mtDNA is relatively high. However, as divergence increases, the chance for multiple transversions at a site increases, thus the Ti/Tv ratio approaches 1:2 when base frequencies are equal and phylogenetic information is eventually lost. Therefore, the simultaneous comparison of divergence and Ti/Tv ratio (as a proxy for saturation) serve as an indicator for phylogenetic information^101^. We calculated the proportion of transitions (%Ti)^101^ to avoid instances where no transversions occurred and compared it with pairwise sequence divergence to assess the potential for saturation. Regions of low %Ti (≤50%)^101^ were defined as likely affected by saturation. We performed the DNA saturation analysis separately for all sites in the protein-coding sequences and also for the first and second codon positions.

### Availability of data

The complete camel mitochondrial genomes obtained in this study are deposited in Genbank with accession numbers listed below: Drom439 (Qatar): KU605072, Drom795 (Saudi Arabia): KU605073, Drom796 (Saudi Arabia): KU605074, Drom797 (Saudi Arabia): KU605075, Drom801A (Austria): KU605076, Drom802 (UAE, Dubai): KU605077, Drom806 (Kenya): KU605078, Drom816 (Sudan): KU605079, Drom820 (Pakistan): KU605080, DC158 (Austria): KU666460, DC269 (Kazakhstan): KU666461, DC399 (Mongolia): KU666462, DC400 (Mongolia):KU666463, DC402 (Mongolia): KU666464, DC408 (Mongolia): KU666465, WC214 (Mongolia): KU666451, WC216 (Mongolia): KU666452, WC218 (Mongolia): KU666453, WC219 (Mongolia): KU666454, WC220 (Mongolia): KU666455, WC247 (Mongolia): KU666456, WC303 (Mongolia): KU666457, WC304 (Mongolia): KU666458, WC305 (Mongolia): KU666459.

## Electronic supplementary material

Supplementary information 
