# Supplementary material for: Mitogenome Sequencing in the Genus Camelus Reveals Evidence for Purifying Selection and Long-term Divergence between Wild and Domestic Bactrian Camels
Source: Sci Rep. 2017 Aug 30;7:9970. doi: 10.1038/s41598-017-08995-8 (PMC5577142; doi:10.1038/s41598-017-08995-8)
Supplement: Supplementary file 1 — Supplementary information [file 41598_2017_8995_MOESM1_ESM.pdf]

Supplementary Information

Mitogenome Sequencing in the Genus *Camelus* Reveals Evidence for Purifying Selection and Long-term Divergence between Wild and Domestic Bactrian Camels

Elmira Mohandesan, Robert R. Fitak, Jukka Corander, Adiya Yadamsuren, Battsetseg Chuluunbat, Omer Abdelhadi, Abdul Raziq, Peter Nagy, Gabrielle Stalder, Chris Walzer, Bernard Faye, Pamela A. Burger

Figure S1: The mtDNA saturation analysis performed on all sites (black) in the protein-coding sequences, and first and second positions (blue). Regions of low %Ti (≤50%) corresponding to sequence divergence of around 0.23 and 0.12 were defined as likely affected by saturation.

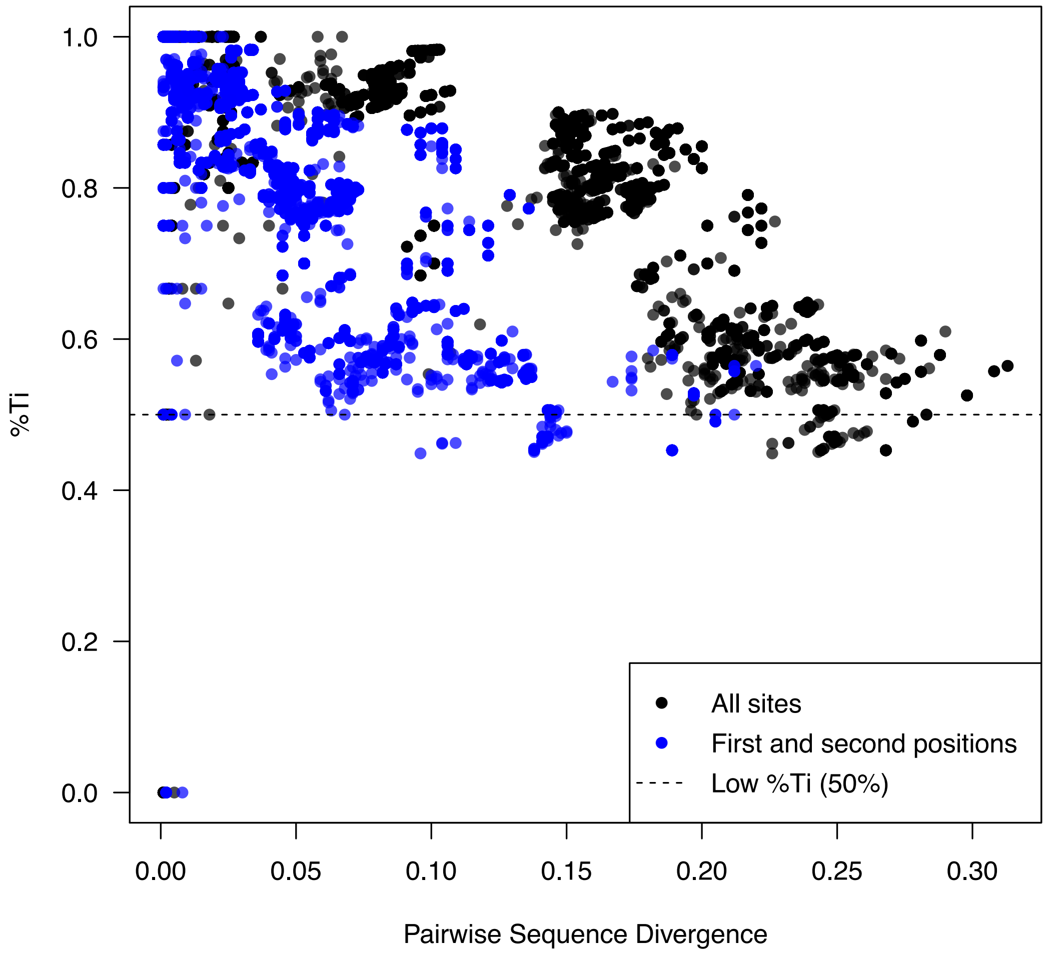

Table S1: List of the 24 mitochondrial genomes of Old world camelids (*C. dromedarius*, *C.bactrianus*, *C.ferus*) obtained in this study. The geographic location of samples, and the length of the recovered mtDNA for each sample are mentioned. The Genbank accession numbers of the mtDNA of 10 different species used in this study are also listed.

Sample ID	Species	Geographical location	GenBank ID	Length (bp)
Dromedary reference	*C. dromedarius*	United Arab Emirates: Dubai	NC_009849.1	16,643
Drom439 [1]	*C. dromedarius*	Qatar	KU605072	16,379
Drom795	*C. dromedarius*	Saudi Arabia	KU605073	16,379
Drom796	*C. dromedarius*	Saudi Arabia	KU605074	16,379
Drom797	*C. dromedarius*	Saudi Arabia	KU605075	16,379
Drom801A [2]	*C. dromedarius*	Austria	KU605076	16,379
Drom802	*C. dromedarius*	UAE, Dubai	KU605077	16,379
Drom806	*C. dromedarius*	Kenya	KU605078	16,375
Drom816	*C. dromedarius*	Sudan	KU605079	16,379
Drom820	*C. dromedarius*	Pakistan	KU605080	16,379
Bactrian reference	*C. bactrianus*	Mongolia, China	NC_009628.2	16,659
DC158 [3]	*C. bactrianus*	Austria	KU666460	16,385
DC269	*C. bactrianus*	Kazakhstan	KU666461	16,385
DC399	*C. bactrianus*	Mongolia	KU666462	16,385
DC400	*C. bactrianus*	Mongolia	KU666463	16,385
DC402	*C. bactrianus*	Mongolia	KU666464	16,385
DC408	*C. bactrianus*	Mongolia	KU666465	16,385
Wild camel reference	*C. ferus*	Mongolia	NC_009629.2	16,680
WC214	*C. ferus*	Mongolia	KU666451	16,383
WC216	*C. ferus*	Mongolia	KU666452	16,383
WC218	*C. ferus*	Mongolia	KU666453	16,384
WC219	*C. ferus*	Mongolia	KU666454	16,383
WC220	*C. ferus*	Mongolia	KU666455	16,384
WC247	*C. ferus*	Mongolia	KU666456	16,384
WC303	*C. ferus*	Mongolia	KU666457	16,383
WC304	*C. ferus*	Mongolia	KU666458	16,383
WC305	*C. ferus*	Mongolia	KU666459	16,384
Llama	*L. glama*		AP003426.1	16,597
Guanaco	*L. guanicoe*		NC_011822.1	16,649
Alpaca	*L. pacos*		Y19184.1	16,652
Vicuna	*V. vicugna*		NC_013558.1	16,084
Cattle	*B. taurus*		AF492351.1	16,338
Tibetan Antelope	*P. hodgsonii*		NC_007441.1	16,498
Tibetan human	*H.sapiens*		KF056318.1	16,569

[1] The individual was sampled in Jordan close to the border to Saudi Arabia, however, its origin from Qatar was confirmed.
[2] The individual's origin can be traced back to North Africa and the Canary Islands.
[3] The individual was sampled in Austria, however, genetically (based on 20 microsatellites) it clustered with Mongolian Bactrian camels.

Table S2: Summary statistics of mitochondrial gene differentiation between dromedaries and domestic Bactrian camels.

Gene	Length (bp)	n	D_a	D_s	D_n
ND1	954	41.1	0.080	68	3
ND2	1041	44.3	0.078	62	15
COX1	1542	44.8	0.063	96	0
COX2	681	27.5	0.077	50	1
COX3	783	29.1	0.069	48	2
AP6	678	28.3	0.078	44	7
AP8	201	10.1	0.093	12	6
CYTB	1137	56.8	0.092	93	9
ND3	348	13.4	0.073	20	4
ND4	1377	61.5	0.081	101	7
ND4L	294	13.6	0.087	24	0
ND5	1818	81.8	0.083	120	24
ND6	525	25.8	0.090	40	6
12S rRNA	967	18.1	0.035	NA	NA
16S rRNA	1563	20.1	0.024	NA	NA
tRNAs	1508	22.5	0.027	NA	NA
CR	953	22.3	0.042	NA	NA
Total	16370	520.9	0.069	778	84

n = average number of nucleotide differences between species; D_a: the number of net nucleotide substitutions per site between species; D_s: number of fixed synonymous substitution between species; D_n: number of fixed non-synonymous substitution between species.

Table S3: Summary statistics of mitochondrial gene differentiation between dromedaries and wild Bactrian camels.

Gene	Length (bp)	n	D_a	D_s	D_n
ND1	954	41.9	0.079	68	4
ND2	1041	44.8	0.077	60	15
COX1	1542	50.9	0.060	90	0
COX2	681	30.3	0.081	52	1
COX3	783	34.0	0.077	55	1
AP6	678	28.2	0.077	44	7
AP8	201	10.5	0.098	12	7
CYTB	1137	61.4	0.099	95	15
ND3	348	13.2	0.070	20	3
ND4	1377	59.3	0.078	97	8
ND4L	294	13.5	0.084	23	0
ND5	1818	85.4	0.084	124	23
ND6	525	27.7	0.097	44	6
12S rRNA	967	20.4	0.038	NA	NA
16S rRNA	1563	20.4	0.023	NA	NA
tRNAs	1508	22.9	0.027	NA	NA
CR	953	23.3	0.043	NA	NA
Total	16370	588.9	0.070	784	90

n = average number of nucleotide differences between species; D_a: the number of net nucleotide substitutions per site between species; D_s: number of fixed synonymous substitution between species; D_n: number of fixed non-synonymous substitution between species.

Table S4: Summary statistics of mitochondrial gene differentiation between domestic and wild Bactrian camels.

Gene	Length (bp)	n	D_a	D_s	D_n
ND1	954	13.6	0.023	20	1
ND2	1041	12.9	0.020	17	2
COX1	1542	12.1	0.014	21	0
COX2	681	7.5	0.018	12	0
COX3	783	10.7	0.022	16	1
AP6	678	6.1	0.016	11	0
AP8	201	2.9	0.025	4	1
CYTB	1137	16.0	0.023	20	6
ND3	348	2.6	0.014	4	1
ND4	1377	18.4	0.022	27	3
ND4L	294	7.3	0.045	13	0
ND5	1818	19.3	0.016	21	7
ND6	525	9.4	0.030	12	3
12S rRNA	967	3.5	0.006	NA	NA
16S rRNA	1563	4.8	0.004	NA	NA
tRNAs	1508	6.0	-0.00005	NA	NA
CR	953	6.8	0.012	NA	NA
Total	16370	160.7	0.018	198	25

n = average number of nucleotide differences between species; D_a: the number of net nucleotide substitutions per site between species; D_s: number of fixed synonymous substitution between species; D_n: number of fixed non-synonymous substitution between species.

Table S5: Pairwise divergence estimates of the electron transport system (ETS) complexes between the three Old world camel species.

ETS complex	mitochondrial genes	Concatenated sequence (bp)	D_a (db)	D_a (df)	D_a (bf)	D_n (db)	D_n (df)	D_n (bf)	D_n (per 1000 bp)		
ETS I	ND1, ND2, ND3, ND4L, ND4, ND5, ND6,	6,357	0.082	0.081	0.022	59	59	17	9.28	9.2	2.6
ETS III	CYTB	1,137	0.092	0.099	0.023	9	15	6	7.91	13.1	5.2
ETS IV	COX1 COX2 COX3	3,006	0.068	0.069	0.017	3	2	1	0.99	0.66	0.33
ETS V	ATP6 ATP8	879	0.081	0.082	0.018	13	14	1	14.7	15.9	1.13

D_a: number of net nucleotide substitutions per site between species; D_n: number of fixed nonsynonymous substitution between species; d: dromedary; b: Bactrian camel and f: wild two-humped camel.

Table S6: Global MK test on 11,379 bp concatenated protein-coding genes conducted on pairwise comparisons between dromedaries, domestic and wild Bactrian camels, respectively. The two-tailed *P*-values of Fisher's exact tests are shown. *P*-values with asterisk are significant with the following significant levels * $P < 0.05$; ** $P < 0.01$.

	Synonymous Substitutions		Non-synonymous Substitutions			
	Fixed differences between species (Ds)	Polymorphic sites among all (Ps)	Fixed differences between species (Dn)	Polymorphic sites among all (Pn)	Neutrality index (NI)	*P*-value
dromedary *vs.* domestic Bactrian	778	125	84	27	2	0.006**
dromedary *vs.* wild Bactrian camel	784	123	90	20	1.41	0.244
domestic *vs.* wild Bactrian camel	198	62	25	17	2.17	0.035*
One- *vs* two-humped camels	678	348	75	57	1.48	0.041*

Table S7: Polymorphisms within *C. dromedarius* species and divergence to *C. bactrianus* at synonymous and nonsynonymous positions in the mitochondrial protein coding genes. The two-tailed *P*-values of Fisher's exact tests are shown. *P*-values with asterisk are significant with significance level of ** $P < 0.01$.

	Fixed synonymous differences (Ds)	Synonymous polymorphic sites (Ps)	Fixed nonsynonymous differences (Dn)	Nonsynonymous polymorphic sites (Pn)	Neutrality index	*P*-value
ND1	68	8	3	1	2.83	0.385
ND2	62	9	15	2	0.91	1
COX1	96	11	0	0	-	1
COX2	50	4	1	0	0	1
ATP8	12	0	6	1	-	0.368
ATP6	44	5	7	1	1.25	1
COX3	48	8	2	0	0	1
ND3	20	3	4	0	0	1
ND4L	24	2	0	0	-	1
ND4	101	15	7	2	1.92	0.352
ND5	120	17	24	4	1.17	0.759
ND6	40	2	6	4	13.33	0.009**
CYTB	93	9	9	0	0	1
Concatenated genes	778	93	84	15	1.49	0.178

Table S8: Polymorphism within *C. dromedarius* and divergence to *C. ferus* at synonymous and nonsynonymous positions in protein coding genes in mtDNA. The two-tailed *P*-values of Fisher's exact tests are shown. The *P*-values with asterisk are significant with significance level of ** $P < 0.01$.

	Fixed synonymous differences (Ds)	Synonymous polymorphic sites (Ps)	Fixed nonsynonymous differences (Dn)	Nonsynonymous polymorphic sites (Pn)	Neutrality index	*P*-value
ND1	68	8	4	1	2.12	0.453
ND2	60	9	15	2	0.88	1
COX1	90	11	0	0	-	1
COX2	52	4	1	0	0	1
ATP8	12	0	7	1	-	0.4
ATP6	44	5	7	1	1.25	1
COX3	55	8	1	0	0	1
ND3	20	3	3	0	0	1
ND4L	23	2	0	0	-	1
ND4	97	15	8	2	1.61	0.629
ND5	124	17	23	4	1.26	0.750
ND6	44	2	6	4	14.66	0.007**
CYTB	95	9	15	0	0	0.600
Concatenated genes	784	93	90	15	1.40	0.249

Table S9: Polymorphism within *C. bactrianus* and divergence to *C. dromedarius* at synonymous and nonsynonymous positions in protein coding genes in mtDNA. The two-tailed *P*-values of Fisher's exact tests are shown. The *P*-values with asterisk are significant with significance level of ** $P < 0.01$.

	Fixed synonymous differences (Ds)	Synonymous polymorphic sites (Ps)	Fixed nonsynonymous differences (Dn)	Nonsynonymous polymorphic sites (Pn)	Neutrality index	*P*-value
ND1	68	3	3	1	7.55	0.200
ND2	62	4	15	1	1.03	1
COX1	96	0	0	0	-	1
COX2	50	0	1	0	-	1
ATP8	12	1	6	1	2	1
ATP6	44	2	7	0	0	1
COX3	48	0	2	2	-	0.004
ND3	20	0	4	0	-	1
ND4L	24	1	0	0	-	1
ND4	101	8	7	1	1.80	0.483
ND5	120	3	24	3	5	0.071
ND6	40	4	6	0	0	1
CYTB	93	6	9	3	5.16	0.056
Concatenated genes	778	32	84	12	3.47	0.001**

Table S10: Polymorphism within *C. bactrianus* and divergence to *C. ferus* at synonymous and nonsynonymous positions in protein coding genes in mtDNA. The two-tailed *P*-values of Fisher's exact tests are shown. The *P*-values with asterisk are significant with significance level of ** $P < 0.01$.

	Fixed synonymous differences (Ds)	Synonymous polymorphic sites (Ps)	Fixed nonsynonymous differences (Dn)	Nonsynonymous polymorphic sites (Pn)	Neutrality index	*P*-value
ND1	20	3	1	1	6.66	0.300
ND2	17	4	2	1	2.12	0.521
COX1	21	0	0	0	-	1
COX2	12	0	0	0	-	1
ATP8	4	1	1	1	4	1
ATP6	11	2	0	0	-	1
COX3	16	0	1	2	-	0.017
ND3	4	0	1	0	-	1
ND4L	13	1	0	0	-	1
ND4	27	8	3	1	1.12	1
ND5	21	3	7	3	3	0.328
ND6	12	4	3	0	0	1
CYTB	20	6	6	3	1.66	0.664
Concatenated genes	198	32	25	12	2.97	0.008**

Table S11: Polymorphism within *C. ferus* and divergence to *C. dromedarius* at synonymous and nonsynonymous positions in protein coding genes in mtDNA. The two-tailed *P*-values of Fisher's exact tests are shown.

	Fixed synonymous differences (Ds)	Synonymous polymorphic sites (Ps)	Fixed nonsynonymous differences (Dn)	Nonsynonymous polymorphic sites (Pn)	Neutrality index	*P*-value
ND1	68	4	4	0	0	1
ND2	60	4	15	1	1	1
COX1	90	3	0	0	-	1
COX2	52	3	1	0	0	1
ATP8	12	0	7	0	-	1
ATP6	44	0	7	0	-	1
COX3	55	4	1	0	0	1
ND3	20	0	3	1	-	0.166
ND4L	23	1	0	0	-	1
ND4	97	2	8	0	0	1
ND5	124	7	23	1	0.77	1
ND6	44	0	6	1	-	0.137
CYTB	95	2	15	1	3.16	0.370
Concatenated genes	784	30	90	5	1.45	0.401

Table S12: Polymorphism within *C. ferus* and divergence to *C. bactrianus* at synonymous and nonsynonymous positions in protein coding genes in mtDNA. The two-tailed *P*-values of Fisher's exact tests are shown.

	Fixed synonymous differences (Ds)	Synonymous polymorphic sites (Ps)	Fixed nonsynonymous differences (Dn)	Nonsynonymous polymorphic sites (Pn)	Neutrality index	*P*-value
ND1	20	4	1	0	0	1
ND2	17	4	2	1	2.12	0.521
COX1	21	3	0	0	-	1
COX2	12	3	0	0	-	1
ATP8	4	0	1	0	-	1
ATP6	11	0	0	0	-	1
COX3	16	4	1	0	0	1
ND3	4	0	1	1	-	0.333
ND4L	13	1	0	0	-	1
ND4	27	2	3	0	0	1
ND5	21	7	7	1	0.42	0.651
ND6	12	0	3	1	-	0.25
CYTB	20	2	6	1	1.66	1
Concatenated genes	198	30	25	5	1.32	0.574

Table S13: Potential positively selected sites detected by branch-site model A implemented in CODEML (PAML) with the correspondent Bayes empirical Bayes (BEB) values. The significance of the detection of sites under positive selection on each of the selected branch was assessed by Likelihood Ratio Test (LRT). n.s: non significant values of LRT. *Posterior probability in BEB > 95%, Posterior probability 50% - 95% without asterisk.

Foreground branch	Gene	Codon	Amino acid	BEB	ΔLRT	d.f	*P*-value
C. dromedarius	ND2	98	V	0.517	0	1	n.s
		219	L	0.504	0	1	n.s
	ATP6	1	V	0.671	0	1	n.s
		38	V	0.592	0	1	n.s
C. bactrianus	ND1	15	V	0.515	-0.46	1	n.s
	ND2	243	L	0.627	0.07	1	n.s
	COX3	41	I	0.543	0	1	n.s
		165	I	0.723	0	1	n.s
	ND3	86	T	0.844	0	1	n.s
	ND4	60	S	0.572	0	1	n.s
	ND5	161	A	0.701	0	1	n.s
		543	T	0.634	0	1	n.s
		592	I	0.697	0	1	n.s
		595	I	0.56	0	1	n.s
C. ferus	ND2	62	T	0.576	0	1	n.s
	ATP8	44	T	0.758	0	1	n.s
	ND4	48	N	0.704	0	1	n.s
		435	A	0.693	0	1	n.s
	ND5	470	N	0.677	0	1	n.s
		495	L	0.678	0	1	n.s
		536	V	0.675	0	1	n.s
	CYTB	4	I	0.638	0	1	n.s
		14	M	0.529	0	1	n.s
		39	V	0.598	0	1	n.s
		349	M	0.618	0	1	n.s
		353	V	0.654	0	1	n.s
Camelus lineage	ND2	227	L	0.649	0	1	n.s
		347	Y	0.625	0	1	n.s
	COX2	94	V	0.589	-0.55	1	n.s
	ATP8	22	V	0.584	0.94	1	n.s
		34	P	0.546	0.94		n.s
		38	S	0.648	0.94		n.s
	COX3	40	S	0.944	2.80	1	n.s
	ND3	19	I	0.537	0.95	1	n.s
	ND4	4	Y	0.514	3.84	1	n.s
		50	L	0.848	3.84		n.s
		82	H	0.953*	3.84		n.s
		183	V	0.918	3.84		n.s
		187	H	0.644	3.84		n.s
		248	L	0.673	3.84		n.s
		438	S	0.508	3.84		n.s
		458	L	0.636	3.84		n.s
	ND5	183	A	0.766	0.11	1	n.s
		432	N	0.838	0.11		n.s

Table S14: Fixed nonsynonymous substitutions between species-specific lineages of camels, their peptide chain locations, inferred ancestral states, and impact on local physicochemical properties are shown. Pairwise comparisons between three camel species (dromedary, domestic and wild Bactrian) are shown with abbreviations db, df and bf. The ancestral states of the amino acid changes were inferred using MEGA6. Magnitude of the amino acid changes (categories ≥ 6) and the affected physicochemical properties are inferred with TreeSAAP (v.3.2): Increase (↑) and decrease (↓) in amino acid properties in a given branch is indicated.

Gene	Amino Acid site	Location	Ancestral state	*C. dromedarius*	*C.bactrianus*	*C.ferus*	Magnitude of change		
							C.dromedarius --> C.bactrianus	*C.dromedarius --> C.ferus*	*C.bactrianus --> C.ferus*
ATP8	36	Nontransmembrane	Ala	Asp	Ala	Ala	7 *** ↓, P_r	7 *** ↓, P_r	
ATP6	47	Nontransmembrane	His	His	Tyr	Tyr	7 *** ↓, α_m	7 *** ↓, α_m	
	51	Nontransmembrane	Arg	Gln	Arg	Arg	7 *** ↑, pHi	7 *** ↑, pHi	
ND3	30	Nontransmembrane	Ile	Thr	Ile	Ile	8 ***↑, R_a<br>8 *** ↑, H_p		
	87	Transmembrane	Thr	Thr	Ile	Thr	8 ***↑, R_a<br>8 ***↑, H_p		8 ***↓, R_a
ND5	69	Nontransmembrane	Ile	Ile	Met	Met		8 ***↑, pK'	
	130	Transmembrane	Ile	Val	Ile	Ile		8 ***↓, pK'	
	162	Nontransmembrane	Ala	Ala	Thr	Ala	6 ***↓, P_α		
	195	Nontransmembrane	Thr	Thr	Ala	Ala	6 ***↑, P_α		
	214	Transmembrane	Met	Ile	Met	Met		8 ***↑, pK'	
	272	Transmembrane	Thr	Met	Thr	Thr	6 ***↓, P_α		
	375	Transmembrane	Ile	Ile	Val	Val		8 ***↑, pK'	
	394	Nontransmembrane	Met	Thr	Met	Met	6 ***↑, P_α		
	398	Nontransmembrane	Ala	Ala	Thr	Thr	6 ***↓, P_α		
	414	Transmembrane	Ile	Val	Ile	Ile		8 ***↓, pK'	

	599	Transmembrane	Ile	Ile	Ala	Ala		8 ***↑, pK'	
ND6	12	Transmembrane	Ile	Ile	Ile	Val		8 ***↑, pK'	
	18	Transmembrane	Ile	Ile	Val	Val		8 ***↑, pK'	
	102	Transmembrane	Ser	Ile	Ser	Ala		8 ***↑, pK'	
	105	Transmembrane	Val	Ile	Val	Val		8 ***↑, pK'	

P_{α}: Alpha-helical tendencies, pK': Equilibrium constant (ionization of COOH), P_r: Polar requirement, H_p: Surrounding hydrophobicity, α_m: Power to be at the middle of alpha-helix, *pHi:* Isoelectric point, R_a: Solvent accessible reduction ratio. The location of the peptides containing the amino acid changes were predicted by the program Phobius. The *P*-values with asterisk are significant with following significant levels *** $P < 0.001$.
